# A promising detection candidate for flagellated *Salmonella* spp.

**DOI:** 10.1186/s13568-019-0851-0

**Published:** 2019-08-14

**Authors:** Yi Yang, Jiangying Zhang, Chunhong Zhu, Xia Meng, Shuhong Sun, Guoqiang Zhu

**Affiliations:** 1 College of Veterinary Medicine, Yangzhou University, Yangzhou, China; 2 Jiangsu Co-Innovation Center for Important Animal Infectious Diseases and Zoonoses, Yangzhou, China; 3 Jiangsu Institute of Poultry Science, Yangzhou, China; 4 College of Animal Science and Technology, Shandong Agricultural University, Tai’an, Shandong China; 5 Joint International Research Laboratory of Agriculture and Agri-Product Safety of Ministry of Education of China, Yangzhou University, Yangzhou, China

**Keywords:** *Salmonella*, Monoclonal antibody (MAb), Flagella, Highly conserved region, Slide Agglutination Test (SAT), Detection

## Abstract

*Salmonella* is a common and important pathogen for both human and animals. All *Salmonella* except *Salmonella pullorum* and *Salmonella gallinarum* have flagellum. Flagellin (FliC) is the main subunit protein forming the bacterial filament, which is present in large amounts on the surface of all flagellated *Salmonella*. After bioinformatics analysis, the most highly conserved region (locates position from 1 to 102 amino acid residue of FliC, we named it as FliC′) was selected, and corresponding recombinant FliC′ (rFliC′) protein was tailored as an immunogen to generate monoclonal antibodies (MAbs) against *Salmonella* flagellin. BALB/c mice were immunized with the purified recombinant protein rFliC′, which were prepared by prokaryotic expression system pET22b (+) expressing FliC′. After fusion of spleen cells from the immunized mice and SP2/0 cells, three hybridoma cells (1D6, 2G6 and 3E2) producing MAbs against targeted flagellate *Salmonella* FliC′ were generated and screened. The ability of MAb 3E2 to recognize and bind to *Salmonella* flagella was demonstrated by immunogold electron microscopy (IEM) method. Western blot (WB) analysis demonstrated that MAb 3E2 could specifically recognize flagellated *Salmonella* strains. Moreover, MAb 3E2 has a direct agglutination activity against *Salmonella* strains with visible agglutination reaction. To further verify this agglutination activity, a total of 52 flagellated *Salmonella* strains (23 serovars), 8 non-flagellate *Salmonella* strains (2 serovars) and 16 other non-*Salmonella* bacteria strains were used to evaluate the specificity of the MAb by direct Slide Agglutination Test (SAT). Results showed that MAb 3E2 reacted with all *Salmonella* strains possessing flagellum and had no cross-reaction with non-flagellate *Salmonella* strains or other non-*Salmonella* bacteria strains. Sequentially, the ability to detect the presence of *Salmonella* in raw samples of the MAb 3E2-based SAT method was evaluated. The conventional culture-based detection method was performed as the standard reference method for detection of *Salmonella*. Altogether, 369 samples collected from laying hens were tested, and the results indicated that the MAb 3E2-based SAT method could specifically detect *Salmonella*. Furthermore, the SAT results were obtained more quickly, as compared with the standard method. As a whole, the MAbs against the tailored conserved region of *Salmonella* flagellin were prepared in this study, and MAb 3E2-based SAT is a promising candidate for the flagellated *Salmonella* spp. rapid detection.

**Electronic supplementary material:**

The online version of this article (10.1186/s13568-019-0851-0) contains supplementary material, which is available to authorized users.

## Introduction

Widely distributed in nature, *Salmonella* are primary enteric pathogen infecting both humans and animals. As one of the most common foodborne pathogen, *Salmonella* cause a variety of symptoms like typhoid fever, gastroenteritis, diarrhea, bacteremia, food poisoning, and so on (Fierer and Guiney 2001; Coburn et al. 2007). So far, more than 2600 *Salmonella* serotypes have been reported, and new serotypes and isolates are constantly being identified (Shi et al. 2015). Therefore, effective and accurate method for *Salmonella* detection is urgently needed for a safe and secure public health.

Currently, *Salmonella* detection methods mainly include microbiological culture, nucleic acid-based technologies and immunoassays (Bell et al. 2016; Cho and Ku 2017). As the traditional ways, bacteria culture and biochemical features tests are still the most widely used methods for *Salmonella* detection. Meanwhile, the methods are limited by moderate sensitivity and time consuming which usually take 7 or more days (Andrews and Ryan 2015). Nucleic acid-based technique (e.g., PCR, qPCR) have provided increased sensitivity and more rapid processing time. However, a complex set of factors can influence the sensitivity and specificity of result, such as the kind of sample, the primers, the target gene, the DNA template quality, and so on (Bell et al. 2016). Immunology-based methods (such as Enzyme Linked Immunosorbent Assay [ELISA]), which are involved in antigen–antibody bindings have been widely used for the detection of food-borne pathogens. MAbs-based immunoassays are essential tools for antigenic characterization and specific detection of various pathogens such as bacteria, virus and parasites (Ghagane et al. 2017; Rohde et al. 2017).

The flagellum of *Salmonella* is a long fibrous structure distributed around the bacteria cell surface. All *Salmonella* species are flagellate with only two exceptions, *Salmonella pullorum* and *Salmonella gallinarum* (Barrow and Freitas Neto 2011). A typical bacterial flagellum consists of three major parts: the basal body embedded in the bacterial cell membrane, the hook functioning as a flexible joint between basal body and filament, and the external filament functioning as a propeller for bacterial locomotion (Aldridge and Hughes 2002; Das et al. 2018). The major function of flagellum is to enable organism possess swimming and swarming motility. Furthermore, flagellum has been demonstrated to act as a virulence factor such as adhesion, invasion, biofilm formation of the pathogen (Parker and Guard-Petter 2001; Duan et al. 2013; Chaban et al. 2015; Barbosa et al. 2017). In addition, the flagellin (filament subunit protein) was considered as a potential target candidate for *Salmonella* detection, since the protein is abundantly expressed, located on the surface of bacteria, and has good antigenicity (Rumbo et al. 2006; Mizel and Bates 2010; Hiriart et al. 2013; Hajam et al. 2017).

In this study, we compared and analyzed amino acid sequence of FliC from different *Salmonella* strains and non-*Salmonella* strains. The most conserved sequence fragments (1–102 aa) of FliC was tailored and prepared for recombinant FliC′ (rFliC′) protein by prokaryotic expression system. The rFliC′ protein sequentially targeted as immunogen to generate MAbs directly recognizing *Salmonella* flagellum. After characterization and analysis, the MAb 3E2 was proved to target the flagella of *Salmonella* in TEM, and the WB result showed that MAb 3E2 could recognize and react with flagellated *Salmonella* strains. Furthermore, we found that the targeted MAb 3E2 possesses a direct and specific agglutination reaction activity with flagellated *Salmonella* strains; and no cross-reactivity with non-flagellate *Salmonella* strains or other non-*Salmonella* bacteria strains. Collectively, the rFliC′ MAb-based agglutination assay was eligible for a rapid, convenient and accurate detection of flagellated *Salmonella* spp.

## Materials and methods

### Plasmids and bacteria strains

The plasmid *p*ET-22b (+) and the *E. coli* DH5α and BL21 (DE3) cells were kept in our laboratory. *Salmonella enteritidis* strain CMCC (B) 50336 (abbreviated as SE50336 in subsequent text) was used as DNA template to PCR-amplify *fliC*′ fragment which was cloned in *p*ET-22b (+) and inducibly expressed in BL21 (DE3) cells for recombinant FliC′ protein production as MAb-producing immunogen and as the positive-control for testing the recognition MAb with *Salmonella* flagella. Additional bacteria used to characterize MAb reactivity and specificity included 52 flagellated *Salmonella* strains, 8 non-flagellate *Salmonella* strains and 16 other non-*Salmonella* bacteria strains. All bacteria strains used in this study were listed in Table 1. SE50336 was kindly offered by Professor Xin’an Jiao in Yangzhou University. *Salmonella typhimurium* T14, U27, A12, W2, W32; *Salmonella paratyphi* O41; *Salmonella typhi* W33, W34, W35; *Salmonella enteritidis* 015, T48, T49, T64; *Salmonella choleraesuis* U80, U81, U82; *Salmonella gallinarum* U20 and *Salmonella arizonae* W36, W37 were kindly offered by Professor Shulin Liu from Harbin Medical University. *Salmonella pullorum* CVCC523, CVCC526, CVCC535, CVCC540 were purchased from China veterinary culture collection center. *E. coli* strains CE2, CE7, CE10 were from Professor Chengping Lu in Nanjing Agricultural University. *Staphylococcus* strains were kindly given by the Institute of Animal Husbandry and Veterinary Medicine, Beijing Academy of Agriculture and Forestry Sciences. The others were kept in our laboratory. All bacteria strains were grown in Luria–Bertani (LB) broth (tryptone [Oxoid, Hampshire, UK] 10.0 g/L, NaCl [Sinopharm Chemical Reagent Co., Ltd, Beijing, China] 10 g/L, Yeast extract [Oxoid, Hampshire, UK] 5.0 g/L) or on LB agar plates at 37 °C.

**Table 1:**
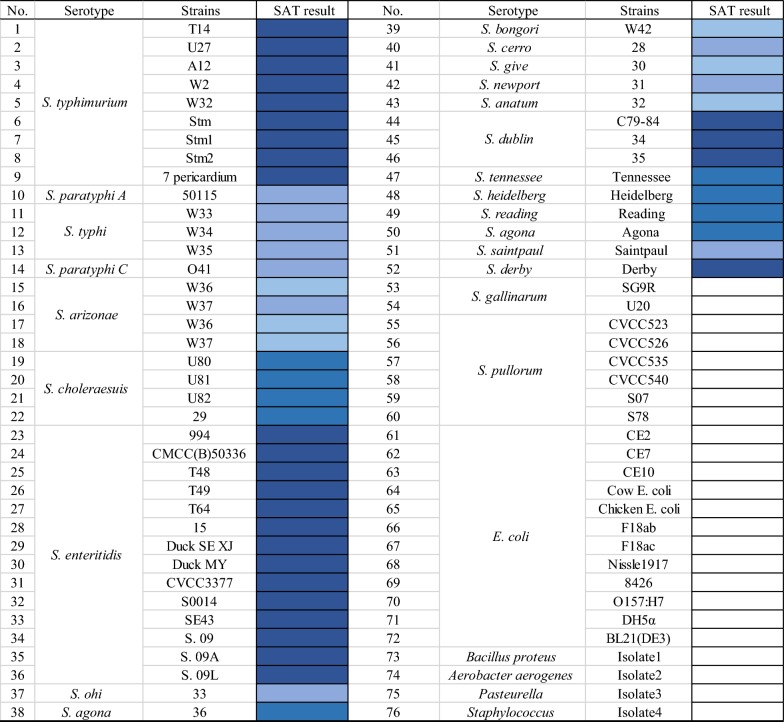
Reactivity and specificity of MAb 3E2 in SAT method

“□” stands for negative reaction, “  ” stands for strong positive, “  ” stands for positive, “  ” stands for mild positive, “  ” stands for slightly positive reaction

### Animals and cells

BALB/c mice (6–8 weeks old, female) were obtained from the Institute of Comparative Medicine of Yangzhou University. The SP2/0 cell line was purchased from the Cell Bank of the Chinese Academy of Sciences (Shanghai, China) and were preserved in our laboratory.

### Sequence analysis of FliC

Nucleic acid sequence of *fliC* from different *Salmonella* strains and non- *Salmonella* strains were acquired from the GenBank database. The predicted amino acid sequences were derived from the DNA sequences using Editseq software in the DNASTAR (version 7.1.0). MEGA 7 software (version 7. 0. 26) was used for phylogenetic analyses by Maximum Composite Likelihood (MCL) method. MegAlign software of DNASTAR was used for amino acid sequence similarity analysis by the Clustal W method. According to the above analysis, highly conserved region of FliC was chose and targeted, and the corresponding purified rFliC′ protein was subsequently used as immunogen to generate MAbs against *Salmonella* flagellin.

### Expression and purification of rFliC′ protein

Expression and purification of rFliC′ were performed using procedures described previously (Yang et al. 2018). Briefly, the fragment *fliC*′ was amplified through PCR directly from the genomic DNA of SE50336 with the forward primer 5′-GCTGCACATATGGCACAAGTCATTAATACAAACAG-3′ and the reverse primer 5′-ACTGCAGCGGCCGCCAACTCACGCACACGCTGCAGGTT-3′ (underline sequences are cleavage sites of restriction enzyme). The amplified *fliC*′ fragment was cloned into the prokaryotic expression vectors *p*ET22b (+) with an N-terminal fusion His-tag. Recombinant plasmid named as *p*ET-*fliC*′ was transformed into *E. coli* BL21 (DE3) by electrotransformation. Under induction of 0.4 mM isopropyl-β-d-thiogalactoside (IPTG, Sigma-Aldrich, St. Louis, USA) at 37 °C 4 h, His-tagged rFliC′ was expressed and existed mostly in form of inclusion bodies. The rFliC′ protein were refolded followed the procedure described in reference (Jungbauer and Kaar 2007). Then, the rFliC′ was purified using a Ni-TED His-tagged protein purification kit (Macherey–Nagel, Germany) according to the instructions of manufacturer.

### Confirmation of rFliC′ by WB

The rFliC′ was identified through WB. Briefly, rFliC′ was electrophoresed on a 15% denatured sodium dodecyl sulfate polyacrylamide gel (SDS-PAGE), followed by transferring onto a nitrocellulose (NC) membrane. After blocking NC membrane with 5% skim milk, the membrane was incubated with anti-His tag antibody (1:1000, Sigma-Aldrich, St. Louis, USA) as primary antibody, followed by incubation with the HRP-labeled goat anti-mouse IgG (1:1500, Sigma-Aldrich, St. Louis, USA) as secondary antibody. The blot was developed using the Diaminobenzidine (DAB) substrate kit (Sigma-Aldrich, St. Louis, USA) according to the instruction manual.

### Generation of MAbs against rFliC′

#### Murine immunization

The immune procedure was followed by a publication previously (Kim et al. 2014). Briefly, four adult female BALB/c mice (8 weeks of age) were subcutaneously immunized with rFliC′ protein (50 μg per mouse) three times with 2 week intervals. Freund’s complete adjuvant (FCA) and Freund’s incomplete adjuvant (FIA) (Sigma-Aldrich, St. Louis, USA) was used to emulsify the rFliC′ protein (1:1; v:v) for the first and subsequent immunizations. At 1 week after the third immunization, serum samples were collected and the antibody titers were tested using indirect ELISA. Three days before hybridoma cell fusion, the mouse with the highest antibody titers was immunized intraperitoneally with rFliC′ (50 μg per mouse) without any adjuvant.

#### Cell fusion and hybridoma selection

Cell fusion and hybridoma selection were performed using the procedure as previously described with a little modification (Ronholm et al. 2011). Briefly, the feeder cells were prepared from the peritoneal cavity of unimmunized BALB/c mouse and seeded into 96-well plates 1 day before fusion. Mouse myeloma cell line SP2/0 were cultured in DMEM medium with 10% FBS (Gibco, USA). Lymphocytes were collected from the immunized mouse spleen, fused with SP2/0 cells at a ratio of 5:1 in the presence of 50% pre-warmed PEG-1500 (Roche, Switzerland). The cell pellet was resuspended in selective HAT medium (Gibco, USA) and cells were seeded into a 96-well plate containing the feeder cells. Ten days after fusion, hybridoma cells were cultured in HT medium (Gibco, USA), and then screened by indirect ELISA. The selected positive clones were sub-cloned by the limiting dilution method. After that, three hybridoma lines (1D6, 2G6 and 3E2) that could secrete MAbs against rFliC′ were obtained and used for further characterizations.

#### Production of ascites

Ascites fluid containing MAbs were produced from cloned hybridoma lines as previously described (Kim et al. 2014). Briefly, BALB/c mice (female, 8 weeks old) were injected intraperitoneally with liquid paraffin (0.1 mL per mouse). One week later, the mice were injected intraperitoneally with 5 × 10^6^ hybridoma cells diluted in PBS. After 10 days, ascites were harvested and purified according to the protocol described in reference (Kim et al. 2014).

### Characterization of MAbs

#### The titer of the MAbs

The hybridoma cell culture supernatants and ascites were collected, then MAbs titers were tested by indirect ELISA following procedure previously described (Zhang 2012). Briefly, each well of plate was coated with 100 μL rFliC′ protein (2 μg/mL) and incubated at 37 °C for 3 h and then 4 °C overnight. Then, the plate was washed 3 times with PBST (PBS containing 0.5% Tween 20). Non-specific binding sites were blocked and incubated with 10% NCS (Gibco, USA) for 2 h at 37 °C. After three times washing, 100 μL of hybridoma supernatants or ascites were added to the wells and incubated 1 h at 37 °C. Then after washing, 100 μL goat anti-mouse IgG conjugated with HRP (1:10,000) was added to each well and incubated for 30 min at 37 °C. Then 100 μL of tetramethylbenzidine (TMB) substrate solution was added into each well and the plate incubated in dark place at room temperature for 2–3 min. The reaction was stopped by adding 50 μL stopping solution (2 M H_2_SO_4_), and the optical density (OD) was measured at 450 nm by an ELISA reader (BioTek, Vermont, USA).

#### Immunogold electron microscopy (IEM)

The ability of the MAbs to recognize and bind the flagella of *Salmonella* was assessed using IEM through a procedure described previously (Ogunniyi et al. 1994). Briefly, SE50336 was grown in LB broth at 37 °C until the optical density of 600 nm up to 1.0. After centrifugation, the cells were resuspended in the same amount of PBS. The samples were subjected to processes of fixation, blocking, incubation with 3E2 MAb (1:10), incubation with colloidal gold-labeled goat anti-mouse IgG (1:50, Sigma-Aldrich, St. Louis, USA), staining, and then observed under a transmission electron microscope (TEM) (Philips Tecnai-12, The Netherlands).

#### Specificity analysis of MAb by WB

The specificity of MAb was analysis by WB described before. Bacterial cell lysates of *Salmonella* strains and non-*Salmonella* strains were transferred to the NC membrane after SDS-PAGE. The membrane was first incubated with the anti-rFliC′ MAbs 3E2 (1:1000) then incubated with secondary HRP-conjugated goat anti-mouse IgG (1:5000). Then detection was performed by using an enhanced chemiluminescence (ECL) substrate kit (Sigma-Aldrich, St. Louis, USA) according to the manufacturer’s protocols.

#### Direct agglutination reaction activity of the MAb 3E2

We revealed that the MAb 3E2 possessed a direct agglutinating ability against SE50336 on slides. In order to verify whether the agglutination activity of MAb 3E2 was also sufficient to other flagellate *Salmonella* strains, we carried out a following test. The specificity of the agglutination reaction was also evaluated at the same time. A number of bacteria strains (Table 1) available in our laboratory collection including 52 flagellated *Salmonella* strains (23 serovars), 8 non-flagellate *Salmonella* strains (2 serovars) and 16 other non-*Salmonella* bacteria strains were used to perform SAT with the MAb 3E2. Agglutination tests procedure were performed as follows: bacteria strains were grown as described previously, washed twice, centrifuged, and resuspended in PBS. MAbs were mixed with the bacterial suspension at a ratio of 1:1 on glass slides. Agglutination reaction was recorded after gentle slide shaking for 1–2 min. For negative controls, the MAb was replaced with PBS.

#### *Salmonella* detection in samples from laying hens in the breed farms

A total of 369 chicken liver and spleen samples were collected from healthy, diseased, or dead laying hen in chicken farms in Jiangsu province. Samples were pre-enrichment for 18 h at 37 °C in buffered peptone water (BPW) followed by a selective enrichment step in selenite cystine (SC) broth for 18 h at 37 °C. Then all the selective enriched samples were detected for the presence of *Salmonella* by SAT using the MAbs 3E2, and the results of agglutination reactions were recorded. All samples was subsequently tested for *Salmonella* through a procedure modified from standard microbiological analysis of China (GB 4789. 4-2010). After confirming on MacConkey (MAC) agar and xylose lysine deoxycholate (XLD) agar, all isolates were serotyped by SAT with a commercial kit of O and H antisera (Tianjin biochip corporation, Tianjin, China).

## Results

### FliC′ (1–102 aa) could be a promising target for *Salmonella* detection

The phylogenetic tree showed that FliC of *Salmonella* had close genetic relationship (Additional file 1: Fig. S1). Similarity analysis of the amino acid sequence of FliC (Additional file 1: Fig. S2) indicated that N-terminal (1–102 aa) region of this protein was highly conserved in *Salmonella*, suggested that FliC′ (1–102 aa of FliC) could be a suitable antigen to MAbs generation for flagellated *Salmonella* detection.

### Preparation and identification of rFliC′

**Fig. 1:**
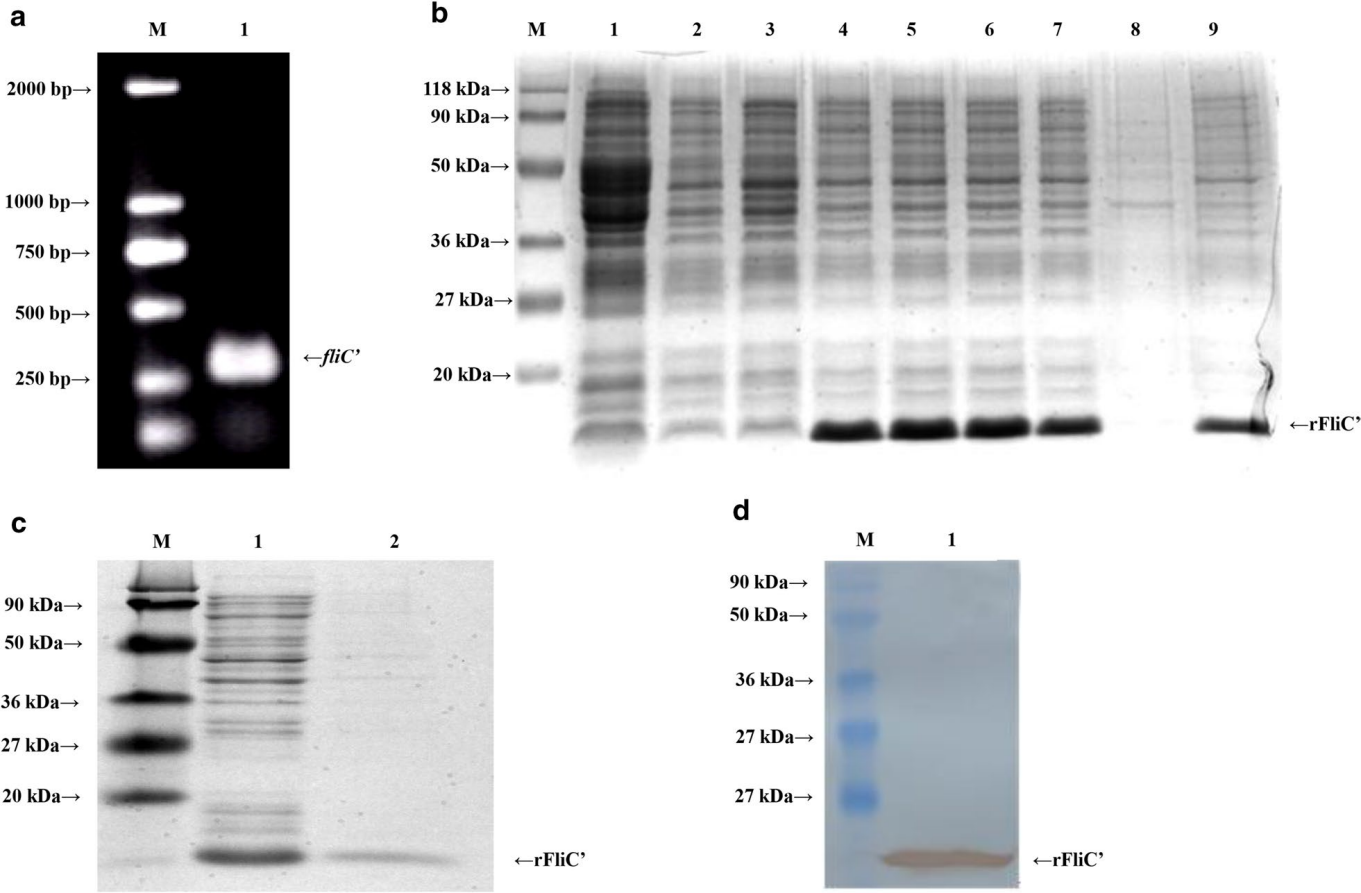
Preparation and identification of recombinant rFliC′. **a** Analysis of *fliC*′ fragment by agarose gel electrophoresis. Lane M, molecular weight marker; lane 1, PCR-amplified *fliC*′ fragment. **b** Detection of the expression of rFliC′ in *E. coli* BL21 (DE3) by SDS-PAGE. Lane M, protein molecular weight marker; lane 1, *p*ET22b (+) vector control; lane 2, BL21 (DE3) control; lane 3–7, cell lysate of *p*ET-*fliC* after IPTG induction (0, 0.1, 0.4, 0.7 and 1.0 mmol/L); lane 8, Cell lysate supernatant of *p*ET-*fliC*; lane 9, cell lysate precipitation of *p*ET-*fliC*. **c** Analysis of the purity of the rFliC′ by SDS-PAGE. Lane M, protein molecular weight marker; lane 1, *p*ET-*fliC* bacterial lysate; lane 2, purified rFliC′ protein. **d** Identification of rFliC′ by WB utilizing anti-His tag antibody. Lane M, protein molecular weight marker; lane 1, protein rFliC′

The *fli*C′ fragment was amplified by PCR from the DNA of SE50336. Two percent agarose gel electrophoresis demonstrated a clear size band about 300 bp consistent with the expected size (308 bp) (Fig. 1a). His-tag fusion protein rFliC′ was expressed in BL21 (DE3) under the induction of 0.4 mM IPTG at 37 °C for 4 h (Fig. 1b). The rFliC′ proteins were purified using Ni^2+^ affinity chromatography column via His-tag, analyzed by SDS-PAGE and WB. The results showed a single band at about 16 kDa of the expected size (Fig. 1c, lane 2) and the recombinant protein could specifically bind to anti-His tag antibody (Fig. 1d).

### Production of anti-rFliC′ MAbs

**Fig. 2:**
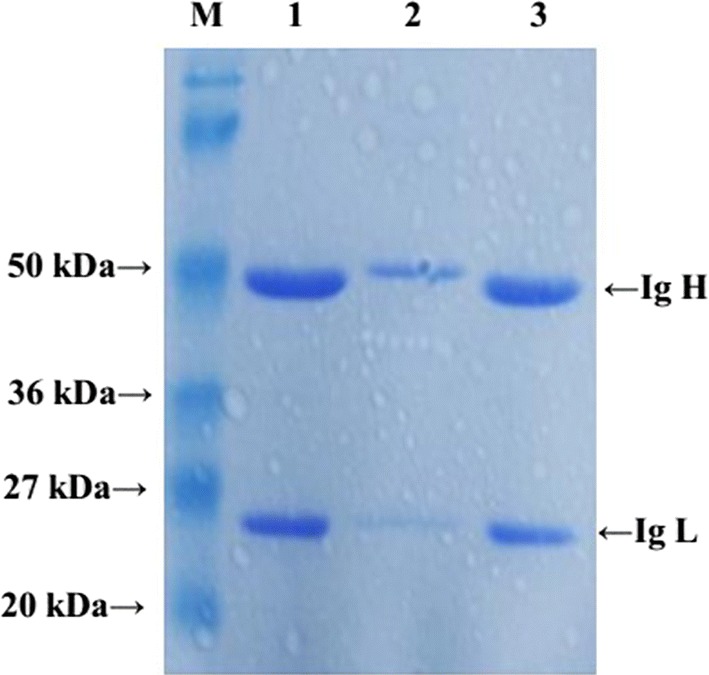
SDS-PAGE analysis for ascites. Bands of 50 kDa and 25 kDa were identified as heavy and light chains. Lane M, protein molecular weight marker; lane 1, mouse ascites of 1D6; lane 2, mouse ascites of 2G6; lane 3, mouse ascites of 3E2

Through the procedures of immunization, fusion, screening and clone selection, three hybridoma cell lines (1D6, 2G6 and 3E2) that stably secreted anti-rFliC′ MAbs were obtained. Ascites were produced from BABL/c mice and purified, two bands corresponding to 50 kDa and 25 kDa (heavy and light chains) were identified with SDS-PAGE gel (Fig. 2).

### The titer of the MAbs

The MAbs titers (1D6, 2G6 and 3E2) in culture supernatants and in ascites were determined by indirect ELISA, and the results were shown in Table 2. The MAb 3E2 with the highest titer in both of cell supernatant and ascites was studied in more details in the subsequent studies.

**Table 2:** MAbs titers of ascites and cell supernatants tested by indirect ELISA

Cell lines	Titers of cell supernatants	Titers of ascites
1D6	5.12 × 10^4^	1.024 × 10^6^
2G6	1.28 × 10^4^	2.56 × 10^5^
3E2	5.12 × 10^5^	1.024 × 10^6^

### IEM

**Fig. 3:**
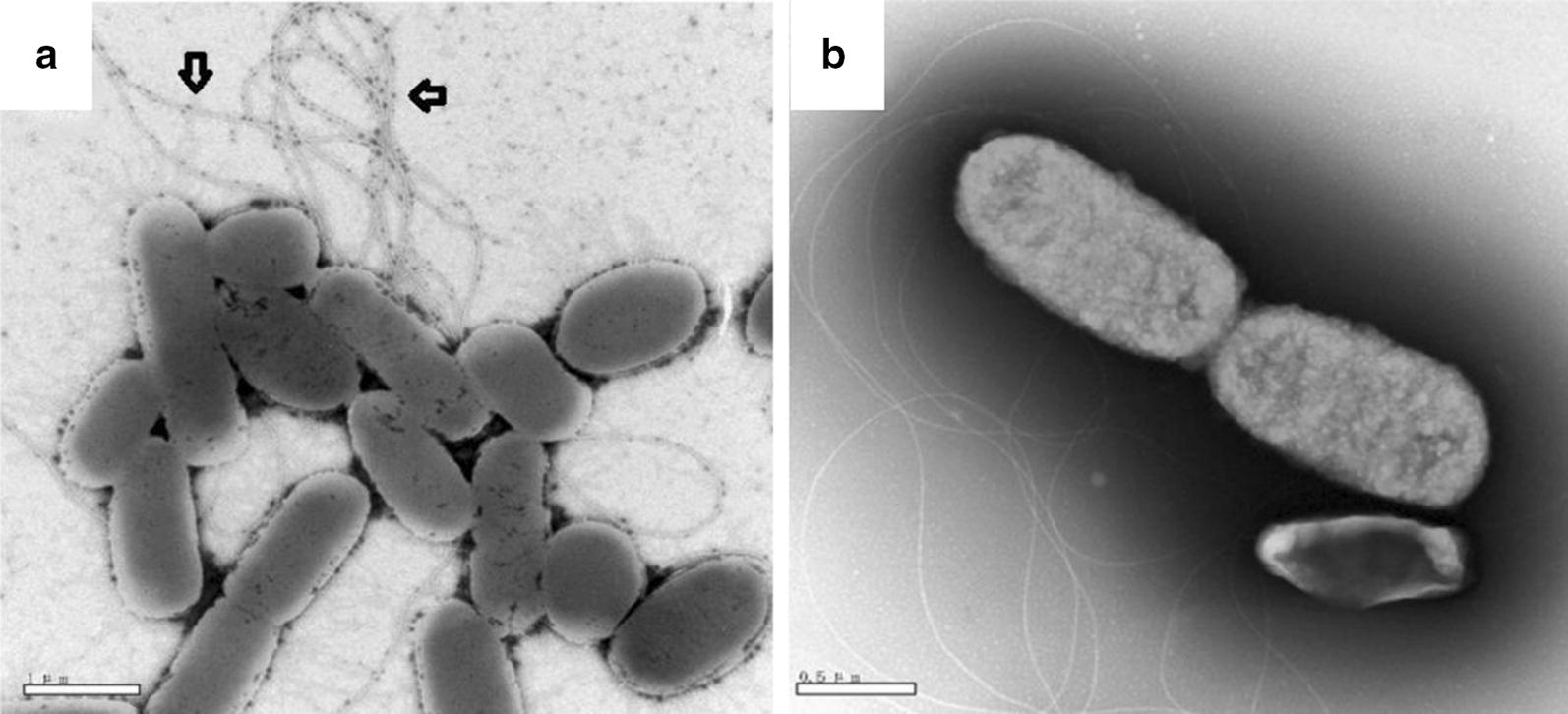
Immunogold electron microscopy analysis of the binding of MAb 3E2 and SE50336 filament. **a** Bacteria cells were incubated with 3E2 MAb followed by reaction with colloidal gold-labelled goat anti-mouse IgG as described in “Materials and methods”. Colloidal gold particles (as the arrow showed) were visualized on the surface of filament demonstrate that 3E2 MAbs were able to recognize and bind to the filament of SE50336. **b** Negative control sample was incubated with same amount of PBS instead of 3E2 MAb

The ability of the MAb to identify *Salmonella* filament was estimated by IEM. The flagellated strain SE50336 was used in the present study. As shown in Fig. 3a, the colloidal gold-labeled antibody was detected clearly at filament flagellar structure of the bacteria (indicated by the arrows), while this phenomenon was not observed in the negative control (Fig. 3b). The result suggested that the MAb 3E2 had the ability to recognize and bind to *Salmonella* with flagella.

### Specificity analysis of the MAb by WB

**Fig. 4:**
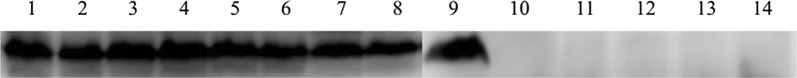
Western blot analysis of specificity of the MAb 3E2. Before test, all the bacteria sample was adjust to the same concentration. Using MAbs 3E2 (1:1000) as primary antibody, and HRP-conjugated goat anti-mouse IgG (1:5000) as secondary antibody. Lane 1–12 were cell lysate of SE50336, 994, T48, W32, Stm1, W36, W37, U81, U82, SG9R, CVCC526, CE2, CE7 and DH5α, respectively. Western blotting results demonstrated that the MAbs 3E2 reacted specifically to the flagellated *Salmonella* strains (lane 1–9). However, no reaction with the non-flagellate *Salmonella* strains (lane 10, 11) or other non-*Salmonella* strains (lane 12–14) was observed

Specificity of the MAb was analysis preliminary by WB using nine flagellated *Salmonella* strains, two non-flagellated *Salmonella* strains and three non-*Salmonella* strains. Result revealed that the MAb was capable to specific detect flagellated *Salmonella* flagellin protein bands with an expected size at 55 KDa, while failed to react with non-flagellated *Salmonella* strains or non-*Salmonella* strains (Fig. 4).

### Specificity evaluation of MAb 3E2-based SAT

**Fig. 5:**
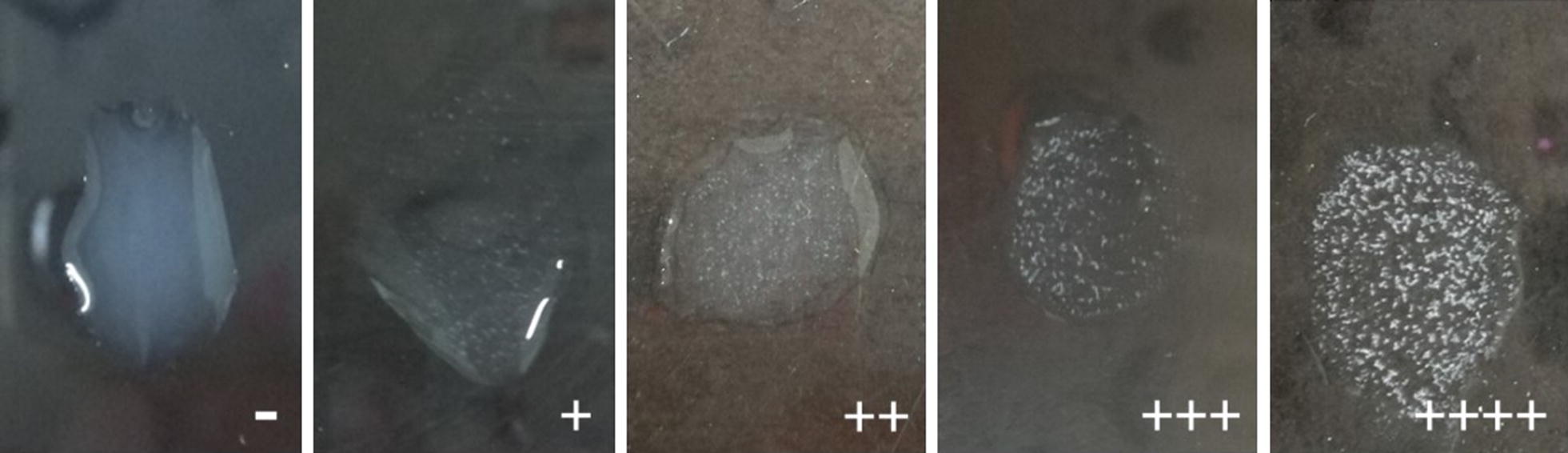
Typical of agglutination assay: negative and positive (from + to ++++) agglutination pattern with 3E2 MAb. “−” stands for negative reaction. “++++”, “+++”, “++” and “+” stands for strong positive, positive, mild positive, slightly positive reaction, respectively

The agglutinating capacities of MAb 3E2 against different bacteria strains were presented in Table 1. The MAbs 3E2 could react with all 52 flagellated *Salmonella* strains. There was no cross-reaction with 8 non-flagellated *Salmonella* strains or 16 non-*Salmonella* strains. Figure 5 presents typical negative and positive reactions. The specific agglutinating capacity of MAb 3E2 against all flagellated *Salmonella* strains indicated this Mab is tremendously promising for flagellated *Salmonella* detection.

### Detection ability in raw samples

Table 3 shows the results for the detection of 369 samples using both the 3E2 MAb-based SAT method and the traditional standard method. After pre-enrichment and selective enrichment (about 40 h), 64 of 389 samples with *Salmonella* were confirmed by 3E2 MAb-based SAT method, 72 of 389 samples were confirmed to be *Salmonella* positive, through traditional microbiological analysis process (about 5 days). The coincidence rate between these two methods was 97.8%. Possessing a high coincidence rate with traditional method and taking less time, the SAT method showed a potential application in specific and rapid *Salmonella* detection. Overall, the 3E2 MAb-based SAT method was confirmed eligible for the specific and rapid *Salmonella* detection.

**Table 3:** Detection ability of FliC′ MAb-based SAT method compared with traditional culture-based method

Methods	Time cost	Positive	Negative	Coincidence rate
FliC′ MAb-based SAT method	40 h	64	305	97.8%
Traditional culture-based method	5 days	72	279	

## Discussion

Since the *Salmonella* spp. is a large set of important zoonotic pathogens, during the last several decades, many methods have been tried for detecting *Salmonella* in kinds of samples during the last several decades (Proux et al. 2002; Shi et al. 2015; Bell et al. 2016; Wang and Salazar 2016). Immunological techniques have a great potential for the detection of *Salmonella* because of their rapidness, sensitivity and specificity. The application of monoclonal antibodies have greatly improved the specificity and efficiency of detection (Ghagane et al. 2017).

*Salmonella* flagellin has good immunogenicity (Mizel and Bates 2010; Chaban et al. 2015) and is constitutively expressed on the bacteria surface. Therefore, flagellin is an obviously candidate antigen target in the detection of *Salmonella*. Nalbantsoy et al. (2010) prepared MAbs against *Salmonella* flagella (H: g, m) by using extracted flagella antigen from *S. enteritidis*. It had been demonstrated that the antibody reacted with H: m and had potential for the diagnosis of *S. enteritidis*, but not good for the diagnosis of other serotypes of *Salmonella*. Wang’s (1993) research groups generated anti-*Salmonella* flagellar monoclonal antibodies in 1993, which used whole bacteria as the immunogen and may result in the following problems, (i) monoclonal antibodies may be generated for different epitopes we can not choose, (ii) screening MAb positive is very difficult and random, (iii) the range of *Salmonella* detection was very limited. In addition, McAb in Wang’s work also had cross reaction with a few of other Enterobacterial strains in the assay.

In our work, we prepared the monoclonal antibody with substantial improvements in both methods and design strategies. First of all, considering there was difference for the choice of immunogen, after bioinformatics analysis, we screened and tailored the FliC′ region with bioinformatics analysis to ensure the conserved region of the *Salmonella* flagellin as the immunogen. It is ensured that the antibody produced is directed against the flagellar conserved region, and this region is present on the surface of the flagellated *Salmonella*. Secondly, for preparation of immunogen, we use the prokaryotic expression system for generation of recombinant protein rFliC′. The use of recombinant rFliC′ as immunogen, instead of whole bacteria or extracted flagellin, ensured that the target antigen was pure and with a good quality which was very important to generate a good antibody in this work. More importantly, in this work, we provide a successful case for the preparation of monoclonal antibodies by using the conserved region of the bacterial surface structure protein as an immunogen.

More notably, we found the MAbs 3E2 have a direct agglutination activity against *Salmonella* strains. Within 76 strains from different bacteria species tested in this work, the 3E2 specifically reacted with flagellated *Salmonella* and did not show any cross-reaction with non-flagellate *Salmonella* strains or other non-*Salmonella* bacteria strains. Agglutination assay utilizing MAbs 3E2 provided a rapid method to detect the presence of flagellated *Salmonella*.

As shown in the results, the MAbs 3E2 was confirmed eligible for the detection of all *Salmonella* strains only with two exceptions of *S. gallinarum* and *S. pullorum* which were non-flagellated. Actually, to overcome this problem and improve the coverage of detection, in our other study, we prepared anti-PegA MAbs based on the following facts. Among 13 major kinds fimbriae operon the *Salmonella* possesses, there is a unique operon termed *peg*, which is so far restricted to *S. gallinarum*, *S. pullorum*, *S. enteritidis* and *S. paratyphi A* (Thomson et al. 2008). The Peg fimbriae present abound on the surface of the bacteria. In addition, it revealed that Peg fimbriae are one of virulence of *Salmonella* since it influences intestinal colonization of chickens (Clayton et al. 2008; Thomson et al. 2008). Therefore, we chose PegA (the major subunit of PEG fimbriae) to serve as an immunogen for generation of anti-PegA MAb, which could specifically recognize *S. gallinarum* and *S. pullorum* (Yang et al. 2018). The anti-PegA MAb was demonstrated to have a good stability and specificity. Using monoclonal antibody 3E2 in combination with the anti-PegA MAbs will benefit a more rapid and convenient method for *Salmonella* detection.

In summary, this report describes the generation and characterization of MAbs specific against the conserved region of *Salmonella* flagellin. This strategy of generating MAbs was workable and efficient. The MAbs 3E2 was demonstrated by IEM that was able to recognize and bind to filament structure of *Salmonella* flagella. In addition, this MAb was confirmed to be capable of specifically detecting flagellated *Salmonella* strains in direct agglutination test. Further, 3E2 MAb-based SAT method was proved eligible for the flagellated *Salmonella* detection in raw samples. Compared with traditional methods, this SAT method is rapid and easy to manipulate, and the entire *Salmonella* detection process could be performed without sophisticated apparatuses or intensive labor. We are looking forward to having a potential application of this MAbs for clinical applications for *Salmonella* detection in the near future.

## Additional file

**Additional file 1: Fig. S1.** Phylogenetic analysis based on predicted amino acid sequences of FliC. **Fig. S2.** Similarity analysis of the amino acid sequence of FliC from different serotype of *Salmonella* strains.

## Data Availability

All data generated or analyzed during this study are included in this published article.
