# Supplementary material for: A promising detection candidate for flagellated Salmonella spp
Source: AMB Express. 2019 Aug 14;9:128. doi: 10.1186/s13568-019-0851-0 (PMC6694378; doi:10.1186/s13568-019-0851-0)
Supplement: Supplementary file 1 — Additional file 1: Fig. S1. Phylogenetic analysis based on predicted amino acid sequences of FliC. Fig. S2. Similarity analysis of the amino acid sequence of FliC from different serotype of Salmonella strains. [file 13568_2019_851_MOESM1_ESM.pdf]

AMB Express

A Promising Detection Candidate for Flagellated Salmonella spp.

Yi Yang[1, 2, 5§], Jiangying Zhang[1, 2, 5§], Chunhong Zhu[3], Xia Meng[1, 2, 5], Shuhong Sun[4*], Guoqiang Zhu[1, 2, 5*]

1. College of Veterinary Medicine, Yangzhou University, Yangzhou, China
2. Jiangsu Co-Innovation Center for Important Animal Infectious Diseases and Zoonoses, Yangzhou, China
3. Jiangsu Institute of Poultry Science, Yangzhou, China
4. College of Animal Science and Technology, Shandong Agricultural University, Tai'an, Shandong, China
5. Joint International Research Laboratory of Agriculture and Agri-Product Safety of Ministry of Education of China, Yangzhou University, Yangzhou, China

Corresponding author 1

Guoqiang Zhu, PhD

E-mail: yzgqzhu@yzu.edu.cn.

Tel: (0086)-514-87972590

Fax: (0086)-514-87311374

Corresponding author 2

Shuhong Sun, PhD

E-mail: ssh6811@163.com

Tel: (0086)-13705389710

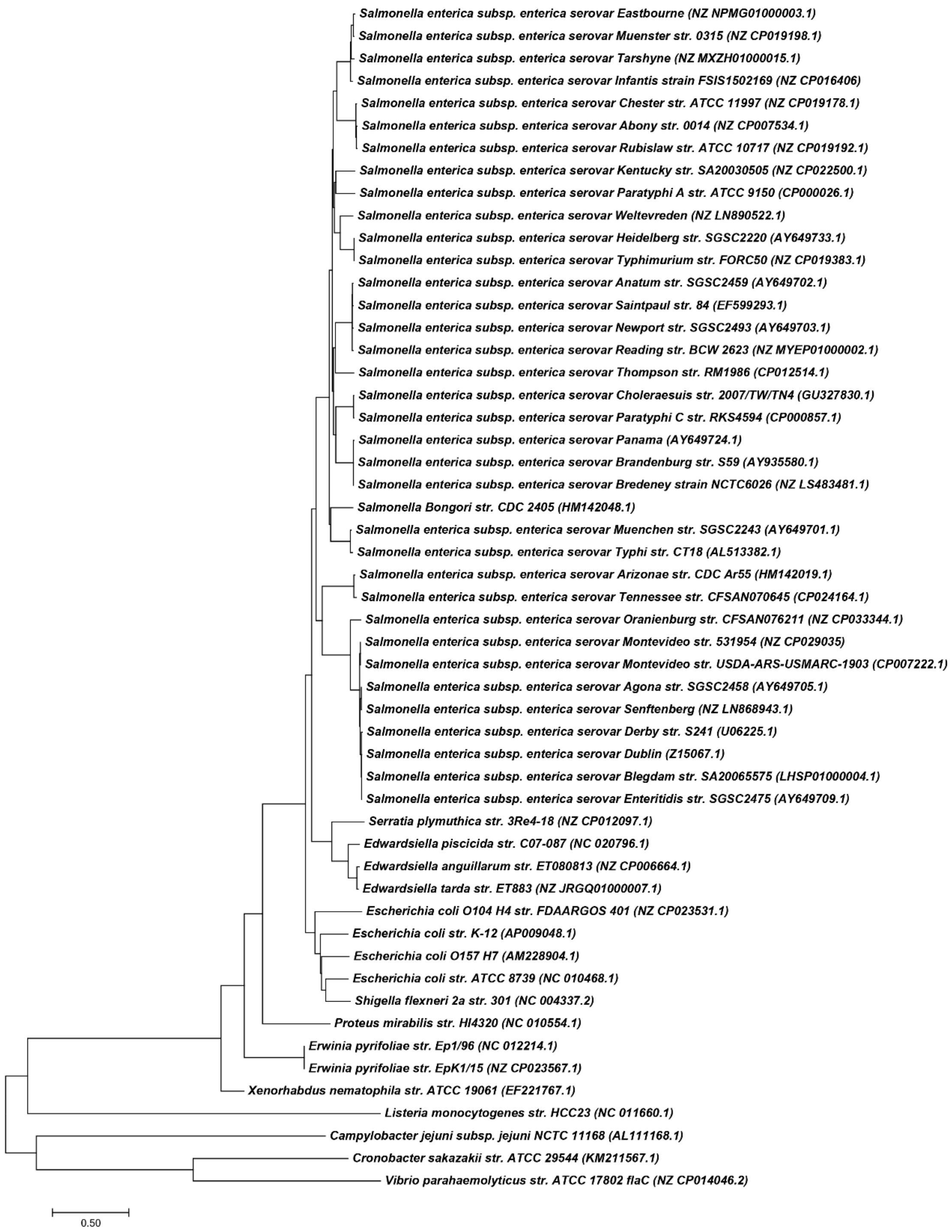

Fig. S1 Phylogenetic analysis based on predicted amino acid sequences of FliC.

The evolutionary distances were computed using the Maximum Composite Likelihood method in MEGA7 (version 7. 0. 26). The analysis involved 53 nucleotide sequences (36 *Salmonella* and 17 non-*Salmonella*). The phylogenetic tree showed that sequences of *fliC* within *Salmonella* species had close genetic relationship.

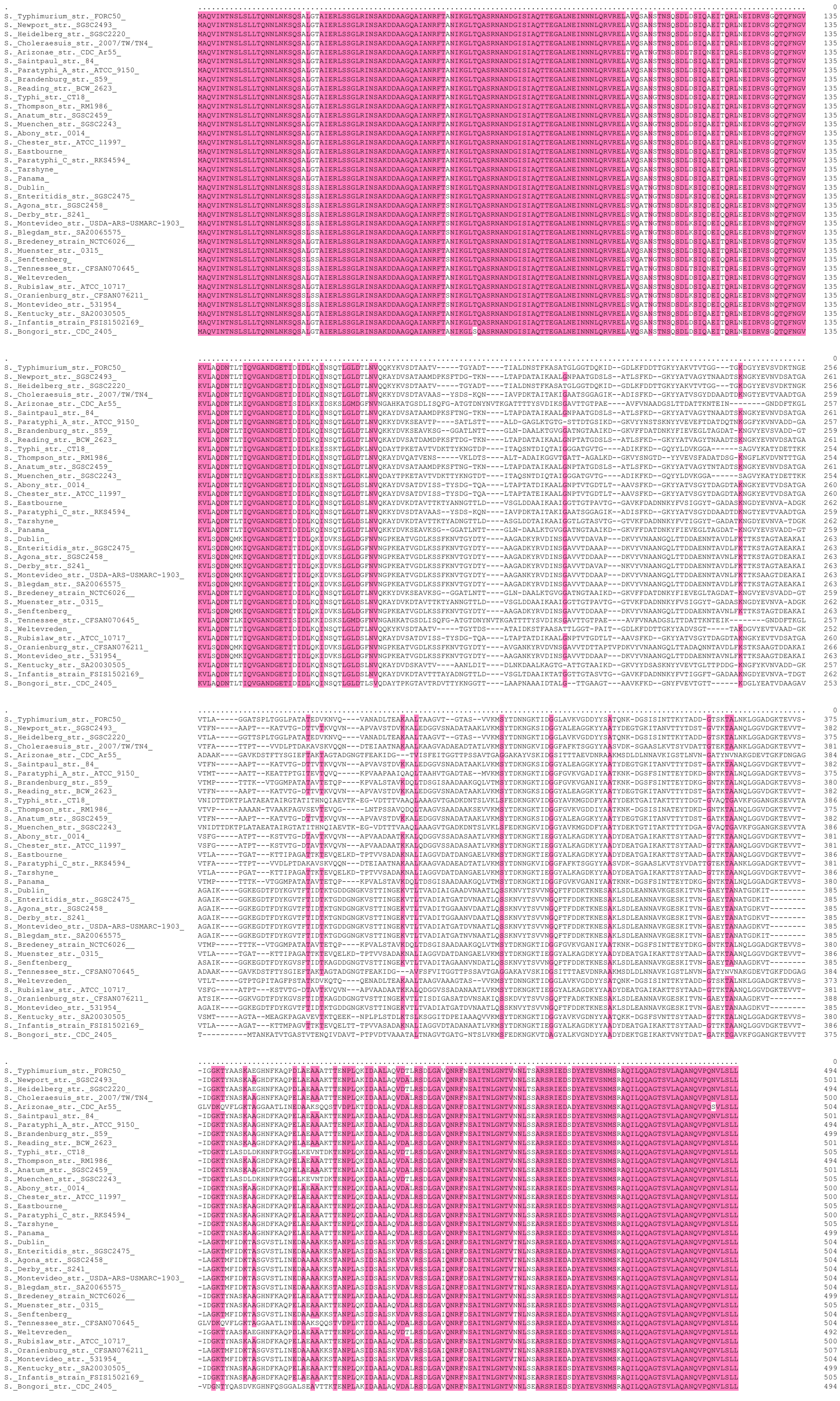
S._Typhimurium_str._FORC50_
S._Newport_str._SGSC2493_
S._Heidelberg_str._SGSC2220_
S._Choleraesuis_str._2007/TW/TN4_
S._Arizonae_str._CDC_Ar55_
S._Saintpaul_str._84_
S._Paratyphi_A_str._ATCC_9150_
S._Brandenburg_str._S59_
S._Reading_str._BCW_2623_
S._Typhi_str._CT18_
S._Thompson_str._RM1986_
S._Anatum_str._SGSC2459_
S._Muenchen_str._SGSC2243_
S._Abony_str._0014_
S._Chester_str._ATCC_11997_
S._Eastbourne_
S._Paratyphi_C_str._RKS4594_
S._Tarshyne_
S._Panama_
S._Dublin_
S._Enteritidis_str._SGSC2475_
S._Agona_str._SGSC2458_
S._Derby_str._S241_
S._Montevideo_str._USDA-ARS-USMARC-1903_
S._Blegdam_str._SA20065575_
S._Bredeney_strain_NCTC6026__
S._Muenster_str._0315_
S._Senftenberg_
S._Tennessee_str._CFSAN070645_
S._Weltevreden_
S._Rubislaw_str._ATCC_10717_
S._Oranienburg_str._CFSAN076211_
S._Montevideo_str._531954_
S._Kentucky_str._SA20030505_
S._Infantis_strain_FSIS1502169_
S._Bongori_str._CDC_2405_

Fig. S2 Similarity analysis of the amino acid sequence of FliC from different serotype of *Salmonella* strains.

The amino acid sequences were aligned by the Clustal W method using the MegAlign of DNASTAR (version 7.1.0). Sites with pink background color represent the conservative region, and sites without background color represent the variable region.
